# Regulating Water Transport Paths on Porous Transport Layer by Hydrophilic Patterning for Highly Efficient Unitized Regenerative Fuel Cells

**DOI:** 10.1007/s40820-025-01684-6

**Published:** 2025-03-17

**Authors:** Sung Min Lee, Keun Hwan Oh, Hwan Yeop Jeong, Duk Man Yu, Tae-Ho Kim

**Affiliations:** Hydrogen Energy Research Center, Korea Research Institute of Chemical Technology (KRICT), 141 Gajeong-ro, Yuseong-gu, Daejeon, 305-600 Republic of Korea

**Keywords:** URFE, Fuel cell, Water electrolysis, Surface modification, Hydrophilic-patterned Ti PTL

## Abstract

While unitized regenerative fuel cells (URFCs) are promising for renewable energy storage, their efficient operation requires simultaneous water management and gas transport, which is challenging from the standpoint of water management. Herein, a novel approach is introduced for examining the alignment hydrophilic pattern of a Ti porous transport layer (PTL) with the flow field of a bipolar plate (BP). UV/ozone patterning and is employed to impart amphiphilic characteristics to the hydrophobic silanized Ti PTL, enabling low-cost and scalable fabrication. The hydrophilic pattern and its alignment with the BP are comprehensively analyzed using electrochemical methods and computational simulations. Notably, the serpentine-patterned (SP) Ti PTL, wherein the hydrophilic channel is directly aligned with the serpentine flow field of the BP, effectively enhances oxygen removal in the water electrolyzer (WE) mode and mitigates water flooding in the fuel cell (FC) mode, ensuring uninterrupted water and gas flow. Further, URFCs with SP configuration exhibit remarkable performance in the WE and FC modes, achieving a significantly improved round-trip efficiency of 25.7% at 2 A cm^−2^.

**Supplementary Information:**

The online version contains supplementary material available at 10.1007/s40820-025-01684-6.

## Highlights

- Novel amphiphilic patterned titanium porous transport layers (PTLs) significantly enhance the round-trip efficiency of unitized regenerative fuel cells (URFCs), achieving an impressive round-trip efficiency of 25.7% at a current density of 2 A cm^-2^.
- The serpentine configuration of the patterned PTL excels in both fuel cell (FC) and water electrolyzer modes, resulting in a sevenfold increase in current density in FC mode compared to URFCs using hydrophilic pristine Ti PTLs.

**Supplementary Information:**

The online version contains supplementary material available at 10.1007/s40820-025-01684-6.

## Graphical abstract

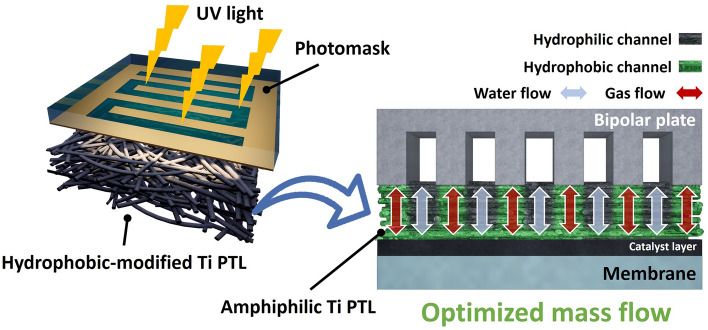

## Introduction

Unitized regenerative fuel cells (URFCs) have emerged as a promising solution for auxiliary energy storage in applications utilizing renewable energy sources [1–6]. URFCs are especially attractive for long-term energy storage because they offer a high theoretical specific energy density without self-discharge [7, 8].

**Fig. 1:**
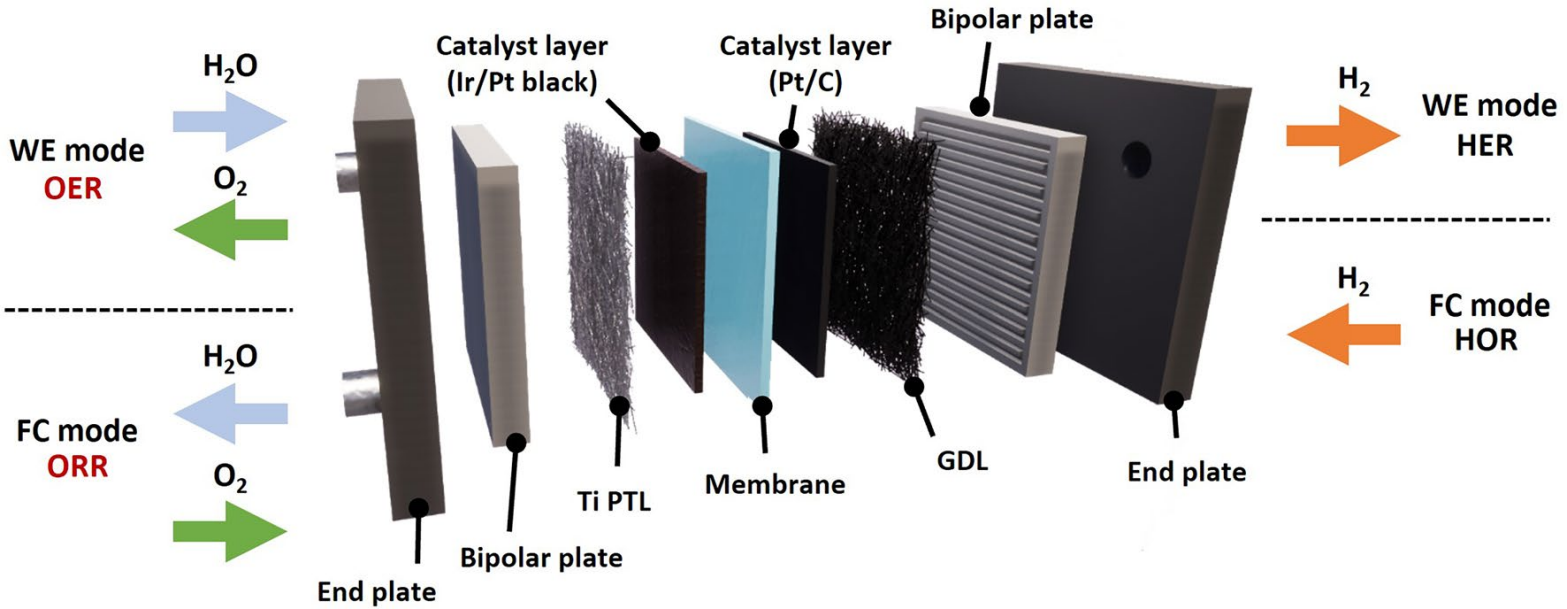
Schematic of the architecture of a URFC

As illustrated in Fig. 1, URFC refers to a single cell with the capability to function in two distinct operation modes, namely the water electrolyzer (WE) and fuel cell (FC) modes. Therefore, the efficient operation of URFCs necessitates simultaneous transport of both hydrophilic water and hydrophobic gas through the cell. However, this inherent contradiction in the round-trip operation poses significant challenges, particularly in the water management in the oxygen electrode, which is crucial for achieving a high round-trip efficiency (RTE) [9].

The porous transport layer (PTL), also referred to as the gas diffusion layer (GDL), plays a crucial role [10] in facilitating water transport from the bipolar plate (BP) to the catalyst layer (CL) in the WE mode and vice versa in the FC mode while ensuring uninterrupted gas transport. To achieve this, carbon-based GDLs, which are conventionally employed in FCs, are used in URFCs [11, 12]. Nevertheless, the hydrophobicity and poor corrosion resistance of the carbon-based GDL remains a fundamental concern that requires further investigation [13].

Consequently, the collective research focus has gradually shifted to the utilization of Ti PTLs, which can withstand the operational voltages used in the WE mode [14]. However, the intrinsic hydrophilicity of Ti PTLs leads to severe water flooding in the FC mode [15, 16]. Thus, considerable efforts have been dedicated to mitigating water flooding in Ti PTLs. For example, many studies have focused on hydrophobically modified Ti PTLs that effectively mitigate water flooding during FC operations [17–22]. However, using hydrophobic modified Ti PTLs also involves a trade-off between the WE and FC performance, particularly at high current densities, owing to their compromised water transport ability.

Because of these persistent challenges associated with water management, recent research has demonstrated the potential of amphiphilic patterned Ti PTLs in facilitating the simultaneous transport of water and gas [23, 24]. This concept holds promise in mitigating the performance trade-off between the WE and FC modes via surface modification. Nevertheless, achieving an optimal RTE in URFCs continues to present a challenge that necessitates a high metal catalyst mass loading (> 2 mg cm^−2^), high oxygen feed rates (2000 sccm for 5 cm^2^), or humidity regulation (65%). Furthermore, the influence of an amphiphilic pattern design on the mass transport associated with the BP and CL has not previously been investigated. Moreover, conventional processes for creating amphiphilic patterns are based on soft lithography, which always presents challenges for cost-effectiveness due to the inherently complex procedures involved [25].

Therefore, in this study, an in-depth analysis of the effects of using hydrophilic patterned Ti PTLs in URFCs was conducted, particularly focusing on their pattern alignment with respect to the flow-field pattern of the BP, regulating water transport paths through the PTL. In addition, the effects of the hydrophilic patterned Ti PTLs on water management were investigated using computational simulations (GeoDict software).

## Experimental Methods

### Fabrication of Hydrophilic Patterned Ti PTL

To modify the surface polarity of the titanium porous transport layer (Pt-coated Ti PTL; Bekaert, Zwevegem, Belgium), 29 mg of 1H,1H,2H,2H-perfluorododecyltrichlorosilane (FDDTS; 729,965, Sigma-Aldrich, USA) was sealed in a jar along with the Ti PTL. Subsequently, a self-assembled layer of FDDTS was deposited on the Ti PTL for 2 h in a preheated 120 °C oven. The Ti PTL coated with FDDTS was exposed to UV light (using a custom UV chamber, Atech LTS, Korea) through a quartz-chrome photomask for 2 h to obtain a hydrophilic patterned Ti PTL.

### Membrane Electrode Assembly (MEA) Preparation

The catalyst was spray-coated on the Nafion 212 (N212, DuPont, USA), which had an active area of 5 cm^2^. This process was conducted on a 90 °C hot plate using a spray coater (LSC-300, Lithotech, Korea) equipped with a nozzle (AccuMist, Sono-Tek, USA). For the anode, a catalyst ink was prepared by mixing Ir black (12,071, Alfa Aesar, USA), Pt black (12,755, Alfa Aesar, USA), Nafion ionomer solution (D521, DuPont, USA), 1-propanol (402,893, Sigma-Aldrich, USA), ethanol (UN1170, Duksan, Korea), and deionized water. The resulting mixture, with a solid content of 0.28%, was sonicated in an ice bath for 60 min with 5 s intervals of rest and sonication using a sonicator (VCX 750, Sonics & materials. Inc., USA). Subsequently, the ink was sprayed at a feed rate of 0.12 mL min^−1^, with the catalyst loading adjusted to 0.7 mg_Ir_ cm^−2^ and 0.3 mg_Pt_ cm^−2^. The ionomer content was 11.6% of the total catalyst loading. For the cathode, the catalyst ink was prepared by mixing Pt/C (TEC10F50E, Tanaka, Japan), Nafion ionomer solution, 1-propanol, and deionized water. The prepared mixture was sonicated in an ice bath for 30 min, with 5-s intervals of rest and sonication. The ink was sprayed at a feed rate of 0.124 mL min^−1^, and the catalyst loading was adjusted to 0.3 mg_Pt_ cm^−2^. The ionomer content was 30% of the mass of Pt/C.

### Unitized Regenerative Fuel Cell (URFC) Test

For the URFC test, the MEA, Ti PTL, carbon gas diffusion layer (3A30, JNTG, Korea), Pt-coated bipolar plate (BP) with a serpentine (SP) flow field, and end plate were assembled together using a torque of 80 kgf cm. Operation of the WE mode was performed using a WE station (PWETS-001, CNL energy, Korea) at 80 °C, ambient pressure, and a flow rate of deionized water of 70 mL min^−1^. For the FC operation, the fuel cell test station (Z010-100, Scitech Korea Inc., Korea) was used under a 45 kPa back pressure at 80 °C and 100% relative humidity with 450 sccm H_2_ flow and 900 sccm air flow.

### Physical Characterization

The surface and cross sectional morphology of MEA was obtained using scanning electron microscopy (SEM; Magna, Tescan, Czech). The surface atomic composition was determined through X-ray photoelectron spectroscopy (XPS; Axis Supra, Kratos, UK). The atomic distribution was mapped using SEM equipped with energy-dispersive X-ray spectroscopy (QUANTAX FlatQUAD, Bruker, USA). Optical images were obtained using high-resolution camera mode of a Samsung Galaxy S22 Ultra and optical microscope (Olympus BX-51, Tokyo, Japan).

### Electrochemical Measurements

Electrochemical characterization was performed using a potentiostat (HCP-803, Bio-logic, USA). Electrochemical impedance spectroscopy (EIS) was conducted over a frequency range of 50 mHz to 100 kHz. Polarization curves were obtained by recording the cell voltage 1 min after each current step. The high-frequency resistance was then obtained by identifying the x-axis intercept at the high-frequency region of the Nyquist plot.

To investigate the electrochemical surface area (ECSA) of the Pt catalyst, cyclic voltammetry was conducted. The hydrogen electrode was supplied with fully humidified 200 sccm H_2_ gas; while, the oxygen electrode was purged with N_2_ gas until the open-circuit voltage dropped below 0.2 V. The voltage was then swept between 70 mV and 1 V at a scan rate of 60 mV s^−1^. The ECSA of the Pt catalyst was calculated based on the area of the peaks in the hydrogen deposition/adsorption regions [26]:

$$ {\text{ECSA}} = \frac{Q}{R \cdot L} $$

where *Q* is the average charge consumed by hydrogen desorption/adsorption, *R* is the charge consumed by the electrochemical conversion of the hydrogen monolayer (210 μC cm^−2^ for Pt), and *L* is the Pt catalyst loading (0.3 mg cm^−2^ in this study).

### Overpotential Analysis

The standard reversible potential for water electrolysis can be obtained from the literature [27]:

$$ E_{{{\text{rev}}}} = 1.2291V - 0.0008456V \cdot \left( {T - 298.15K} \right) $$

In this study, $E_{{{\text{rev}}}}$ was calculated to be 1.18 V under the 80 °C operation condition, and each overpotential term was calculated using the following equations:

$$ {\text{WE mode}}:E_{cell} = E_{rev} + \eta_{kin} + \eta_{ohm} + \eta_{mass}  $$

$$ {\text{FC mode}}:E_{{{\text{cell}}}} = E_{{{\text{rev}}}} - \left( {\eta_{{{\text{kin}}}} + \eta_{{{\text{ohm}}}} + \eta_{{{\text{mass}}}} } \right) $$

where $\eta_{{{\text{kin}}}}$ is the kinetic, $\eta_{{{\text{ohm}}}}$ is the Ohmic, $\eta_{{{\text{mass}}}}$ mass transport overpotential (also referred as voltage loss for FC). First, for $\eta_{{{\text{kin}}}}$, both HER and HOR are more favorable than OER and ORR, and $\eta_{{{\text{kin}}}}$ is predominantly governed by OER and ORR. $\eta_{{{\text{kin}}}}$ can then be approximated from the Tafel equation based on the polarization curve obtained in the low current density region ($i$ ≤ 100 mA cm^−2^) where the overpotential is predominantly governed by a charge transportation process [28].

$$ \eta_{{{\text{kin}}}} = b \cdot {\text{log}}\left( {\frac{i}{{i_{0} }}} \right) $$

where *b* is the Tafel slope,$$ i_{0}$$ is the exchange current density, and $$i$$ is the current density of the cell. Second, the $$\eta_{{{\text{ohm}}}}$$ can be calculated by $$\eta_{{{\text{ohm}}}} = i \cdot {\text{HFR}}$$, where the *HFR* is high-frequency resistance at the x-axis intercept obtained from the Nyquist plot. $$\eta_{{{\text{mass}}}}$$ can then be calculated using the following relationships:

$$ {\text{WE mode}}:\eta_{{{\text{mass}}}} = E_{{{\text{cell}}}} - \left( {E_{{{\text{rev}}}} + \eta_{{{\text{kin}}}} + \eta_{{{\text{ohm}}}} } \right) $$

$$ {\text{FC mode}}:\eta_{{{\text{mass}}}} = E_{{{\text{rev}}}} - \left( {E_{{{\text{cell}}}} + \eta_{{{\text{kin}}}} + \eta_{{{\text{ohm}}}} } \right) $$

### Computational Study with GeoDict

The modeling domain was designed as a cubic space to represent both solid and void regions, featuring a realistic morphology intended to mimic the wire structure of a PTL. These PTLs are typically composed of Pt-coated Ti wires, each having a diameter of approximately 50 μm. The Ti wires are assumed to have a constant cylindrical shape and extend infinitely in the x–y plane within the various geometries. To create a manageable microstructure model, we disregarded any interactions between the Ti wires and ensured that they did not overlap. The geometric characteristics of the microstructure, such as porosity, radius, and directional distribution, were determined based on the parameters of the model to validate the 3-D structure of the PTL, which was generated using the FiberGeo modules available in the GeoDict software.

The static distribution of wetting and non-wetting phases under varying capillary pressures can be assessed using a pore morphology technique. This method is applicable for analyzing the distribution of these two phases during a drainage process or for repeated cycles of drainage and imbibition. To calculate the typical distribution of the non-wetting phase, in this case, air, across the diverse pore structure within the PTL, we used a numerical solver module called SatuDict within the GeoDict software.

## Results and Discussion

### Fabrication of Water Transport Regulating Ti PTL

**Fig. 2:**
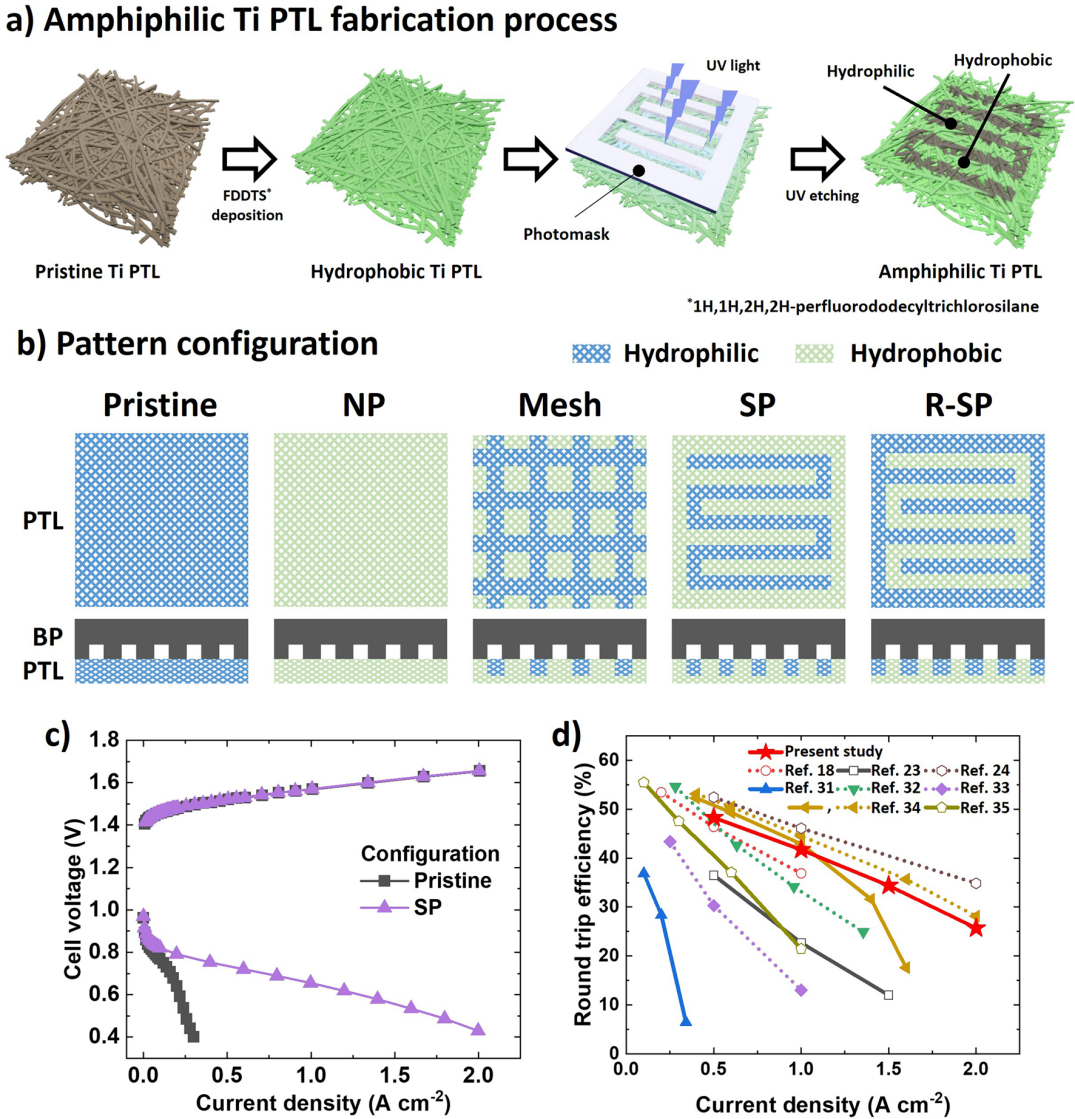
**a** Schematic of the fabrication process for hydrophilic patterned Ti porous transport layers (PTLs). **b** Pattern configuration and alignment with respect to the serpentine (SP) flow field bipolar plate. **c** Polarization curves of the pristine and SP configurations. **d** Comparison of round-trip efficiencies. The dashed and solid lines indicate the oxygen and air feeds in the fuel cell (FC) mode, respectively. The empty and filled symbols indicate high (> 2 mg cm^−2^) and low (< 2 mg cm^−2^) catalyst loadings, respectively

The fabrication of various hydrophilic patterned Ti PTLs was realized using a UV/ozone patterning method (Figs. 2a, S1 and Note S1) combined with hydrophobic silanization of Ti PTL [29, 30]. Remarkably, among the five configurations examined in this study (Fig. 2b), the serpentine configuration (SP) stands out due to its hydrophilic flow channels, which are aligned with the flow field of the bipolar plate (BP). Remarkably, among the five configurations examined in this study (Fig. 2b), the SP stands out due to its hydrophilic flow channels, which are aligned with the flow field of the BP. SP configuration not only demonstrated excellent fuel cell (FC) performance but also water electrolysis (WE) performance, with highest RTE of Pristine: N/A, NP: 40.4%, Mesh: 39.5%, R-SP: 41.1%, and SP: 41.7% at 1 A cm⁻^2^. Compared with the hydrophilic pristine configuration, (0.28 A cm^−2^ @ 0.43 V), the SP exhibited a seven-times higher current density (2.0 A cm^−2^ @ 0.43 V) in the FC mode (Fig. 2c). Moreover, the URFCs reported in this study are the first to achieve a successful round-trip operation up to 2 A cm^−2^, which was facilitated by an air feed in the FC mode with a small catalyst loading of 1.3 mg cm^−2^. Our URFC shows an impressive RTE improvement up to 25.7% at 2 A cm^−2^, and its performance is competitive with those reported in previous studies (Fig. 2d and Table S1) [18, 23, 24, 31–35]. This finding highlights the crucial role of regulating water transport path in PTL in achieving efficient water and gas transport during the round-trip operation of URFCs.

As introduced in Fig. 2b, five distinct configurations were designed to investigate the effect of the hydrophilic pattern on the mass transport between the BP and the Ti PTL. Specifically, three pattern designs were investigated and compared with the pristine and hydrophobic non-patterned (NP) fluorosilanized Ti PTLs. The mesh design (Mesh) incorporated a hydrophilic pattern arranged in a mesh shape with a line width and spacing of 1 and 2.34 mm, respectively, while the remaining square area was rendered hydrophobic. Both the SP and reverse serpentine (R-SP) designs featured a hydrophilic line width and spacing of 1 and 1.1 mm, respectively. However, in the SP, the area in contact with the flow field of the BP became hydrophilic, while that in the R-SP became hydrophobic. In addition, the areal ratios of the hydrophilic channels of each pattern were similarly adjusted. Specifically, the Mesh, SP, and R-SP designs were adjusted to 45%, 49%, and 46%, respectively, to minimize the influence of the areal ratio between the hydrophilic and hydrophobic channels.

**Fig. 3:**
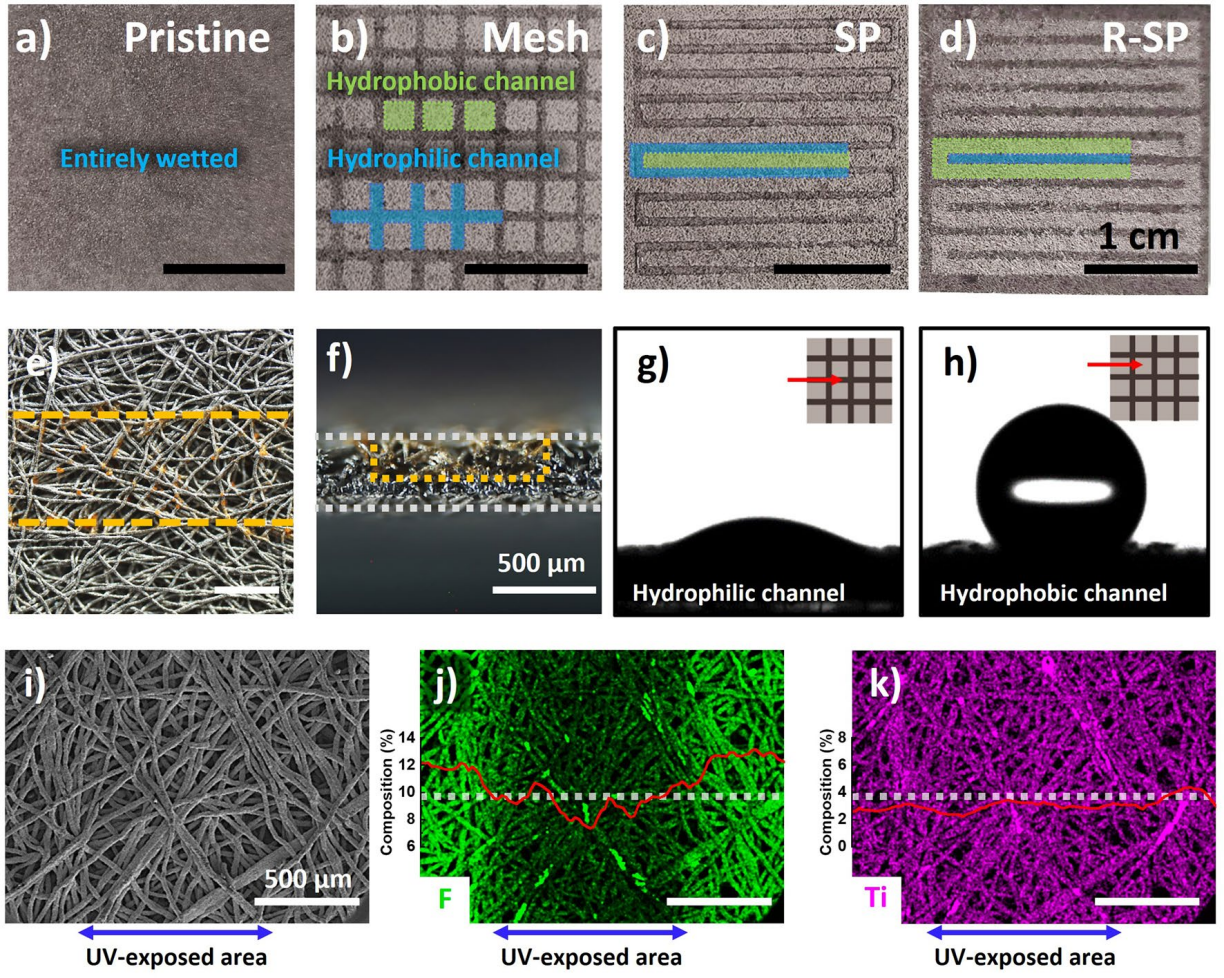
**a–d** Optical images of a Ti PTL after water droplet deposition. The **e** top and **f** cross sectional views of the microscope images of the SP Ti PTL after being stained with a 1 wt% methyl orange solution. The yellow line highlights the hydrophilic channel area. The water contact angle of the **g** hydrophilic and **h** hydrophobic areas of the channel in the Mesh Ti PTL. **i** SEM images, along with the corresponding energy-dispersive X-ray spectroscopy (EDS) mapping and line scan results, of the **j** F and **k** Ti distributions in the SP Ti PTL. The dashed line indicates the direction of the line scan

Figure 3a–d provides a visual distinction between the wettabilities of the pristine and hydrophilic patterned Ti PTLs. The hydrophilic channel was visualized by staining with a methyl orange solution, based on dewetting-driven micropatterning technique [36]. This visualization highlighted that the hydrophilic channel extended to a depth of 220 μm from the surface and had a water contact angle of 27.7° and contact angle of 126° (Fig. 3e–h). This significant polarity contrast contributed to the development of the amphiphilic characteristic, which was crucial for regulating the water transport path, separating from gas flow channels (Movies S1 and S2). In addition, the energy-dispersive X-ray spectroscopy (EDS) mapping images revealed a distinctive gradient of fluorine composition across the UV-exposed area (Fig. 3i–k).

### Electrochemical Analysis in WE Mode

**Fig. 4:**
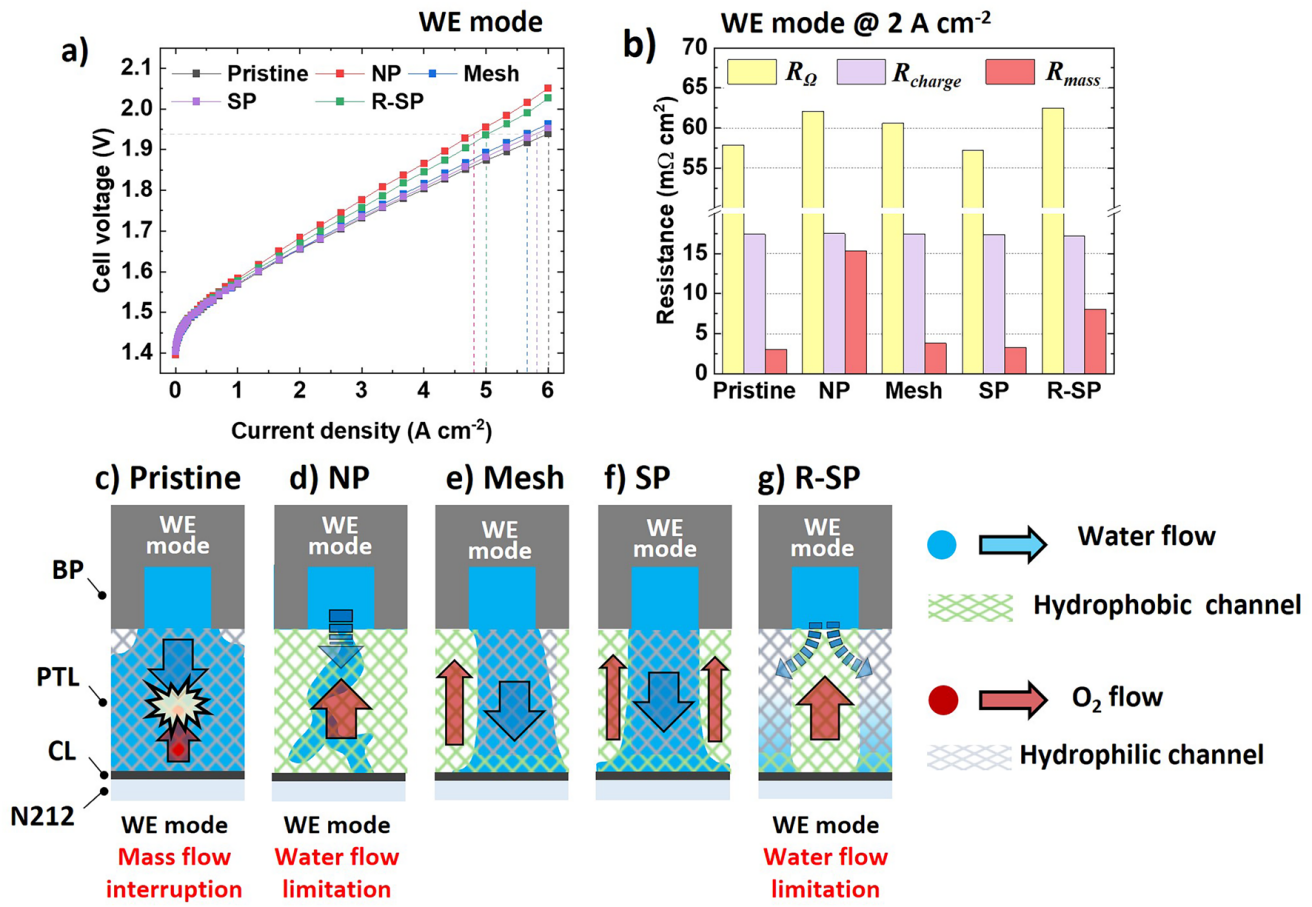
**a** Polarization curves with various pattern configurations in the WE mode and **b** the corresponding resistance contributions obtained from Nyquist plot at 2 A cm^−2^. Illustration of mass flow in the WE mode for **c** Pristine, **d** NP, **e** Mesh, **f** SP, and **g** R-SP

Subsequently, a comprehensive electrochemical analysis was conducted to understand how each pattern configuration influenced mass transport during the round-trip operation of the URFCs. Notably, when the hydrophobic channels of a PTL come into direct contact with the flow field of the BP in the WE mode (Fig. 4a), a significant decrease in the current density is observed. This was confirmed by comparing the current densities of the NP (4.80 A cm^−2^ @ 1.94 V) and R-SP (5.05 A cm^−2^ @ 1.94 V) systems to that of the pristine configuration (6.00 A cm^−2^ @ 1.94 V). The corresponding Nyquist plot (Fig. S2) and resistance contribution (Fig. 4b) also indicate that the mass transport resistances (${R}_{\text{mass}}$) of the NP and R-SP (15.35 and 8.05 mΩ cm^2^, respectively) were significantly higher than that of the pristine configuration (3.05 mΩ cm^2^). This result suggests that when the water feed from the BP comes into contact with the hydrophobic channel, it slowly diffuses toward the hydrophilic channel and CL [37, 38]. Furthermore, the NP and R-SP exhibited higher Ohmic resistances (${R}_{\Omega }$) of 62.05 and 62.50 mΩ cm^2^, respectively, compared with that of the pristine configuration (${R}_{\Omega }$ = 57.85 mΩ cm^2^). This result implies that the electrolyte content in the NP and R-SP decreased because of their small water fluxes, which in turn increased the number of vacancies (Fig. 4d, g) [39–41].

In contrast, when the hydrophilic channel in the PTL is partially (Mesh) or properly aligned (SP) to the flow field of the BP, water can easily be transported toward the CL, enabling efficient mass transport, as seen in the Mesh (5.67 A cm^−2^ @ 1.94 V) and SP (5.82 A cm^−2^ @ 1.94 V). In addition, the Mesh and SP exhibited ${R}_{\text{mass}}$ values of 3.80 and 3.25 mΩ cm^2^, respectively, which are comparable to that of the pristine configuration. This result is notable considering that the hydrophilic channel areas of the Mesh and SP are only half the size of that in the pristine configuration; that is, higher water flow velocities are developed within the Mesh and SP owing to the minimized interruptions between the oxygen and water flow (Fig. 4e, f), whereas in the pristine configuration, the water flow is interrupted by the oxygen flow (Fig. 4c).

### Electrochemical Analysis in FC Mode

**Fig. 5:**
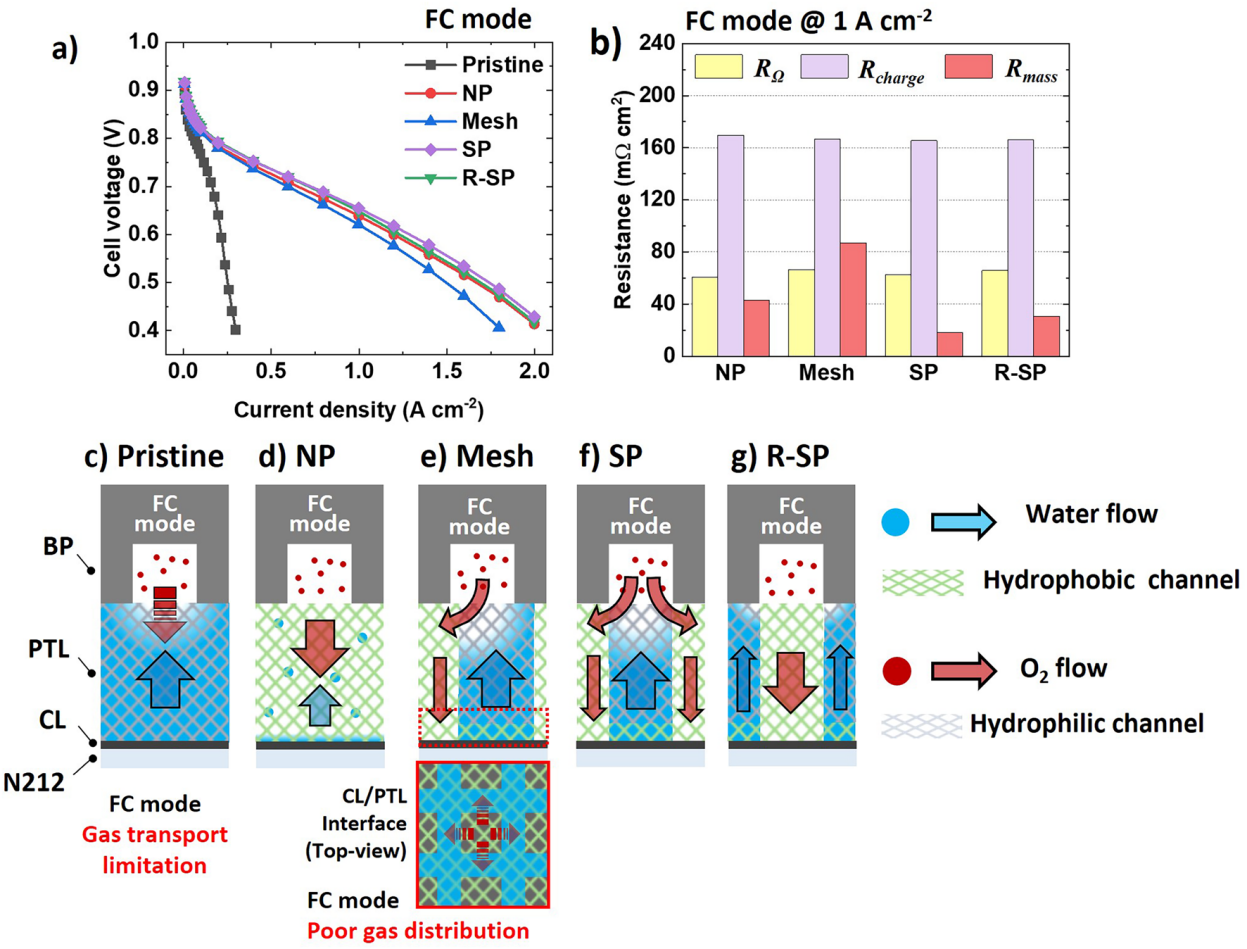
**a** Polarization curves with various pattern configurations in the FC mode and **b** the corresponding resistance contributions obtained from the Nyquist plot measured at 1 A cm^−2^. Illustration of mass flow in the FC mode for the **c** Pristine, **d** NP, **e** Mesh, **f** SP, and **g** R-SP configurations (Nyquist plot of Pristine configuration at 1 A cm^−2^ was not measurable current density limit)

In the FC mode, the cell voltage of the pristine configuration exhibited a steep drop to 0.43 V at 0.28 A cm^−2^ (Fig. 5a). As shown in the corresponding Nyquist plot and resistance contribution in Fig. S3a and b, the use of the pristine configuration leads to a significant increase in both the ${R}_{\text{charge}}$ and ${R}_{\text{mass}}$ to 2320 and 370 mΩ cm^2^ at 0.3 A cm^−2^, respectively, which are an order of magnitude larger than other configurations. The steep voltage drop observed can be attributed to water flooding in the fully hydrophilic pristine configuration. This flooding disrupts the mass transport of oxygen to the catalyst layer (CL), significantly reducing the effective surface area of the catalyst (Fig. 5c) [42]. With the catalyst surface area compromised, the rate of oxygen reaching the Pt surface diminishes, which is critical for efficient electrochemical reactions. Moreover, the limited solubility of oxygen in water further decreases the availability of oxygen for the reactions occurring on the Pt surface [43]. Consequently, as the current density increases, both activation and mass transport voltage losses associated with the pristine configuration exhibit a steep voltage drop in polarization curve (Fig. S4d, f).

However, all surface-modified configurations exhibited a notable increase in their current densities. In addition, the implanted hydrophobic flow channel significantly reduced both ${R}_{\text{charge}}$ and ${R}_{\text{mass}}$ (Figs. 5b and S3b). Nevertheless, among all the surface-modified configurations, the cell voltage of the Mesh showed the steepest decrease (Fig. 5a). Notably, the Mesh exhibited the highest ${R}_{\text{mass}}$ of 87.00 mΩ cm^2^. Furthermore, as the current density increased, the Ohmic and mass transport voltage losses of the Mesh increased significantly compared with those of the other patterned configurations (Fig. S4e, f). This result indicates that the in-plane oxygen distribution with the Mesh is relatively restricted to a square-shaped enclosed hydrophobic channel owing to the water flood surrounding hydrophilic channel near the CL (further details are discussed in Note S2 and Sect. 3.4 with flow dynamics simulations based on GeoDict). Consequently, hot spots develop under the hydrophobic channel area, and the membrane is more likely to become dehydrated, which results in an increase in Ohmic loss [44]. Among the NP, SP, and R-SP, ${R}_{\text{mass}}$ decreased in the following order: NP > R-SP > SP. This suggests that the hydrophilic channels in the SP (Fig. 5f) and R-SP (Fig. 5g) played a role in removing water and preventing water saturation in the CL, compared to the NP (Fig. 5d) [45]. In addition, the ${R}_{\text{mass}}$ value was minimized to 17.97 mΩ cm^2^ in the SP, even though its hydrophilic channel was directly aligned with the flow-field pattern of the BP. This result is obtained because unlike liquids, the diffusivity of gases is less sensitive to the surface polarity of the medium and is more influenced by the porous structure of the medium [46–48]. In addition, compared with the hydrophilic channel near the CL, the water was drained toward the flow field of the BP, thus creating pores for gas diffusion in the hydrophilic channel near the BP. Consequently, in the FC mode, even when the hydrophilic channel was directly aligned with the flow-field pattern of the BP, the gas easily flowed through the hydrophilic channel and reached the hydrophobic gas channel. Therefore, the SP enabled efficient mass flow of both water and gas by effectively regulating water transport path in Ti PTL.

### Flow Dynamics Simulation

**Fig. 6:**
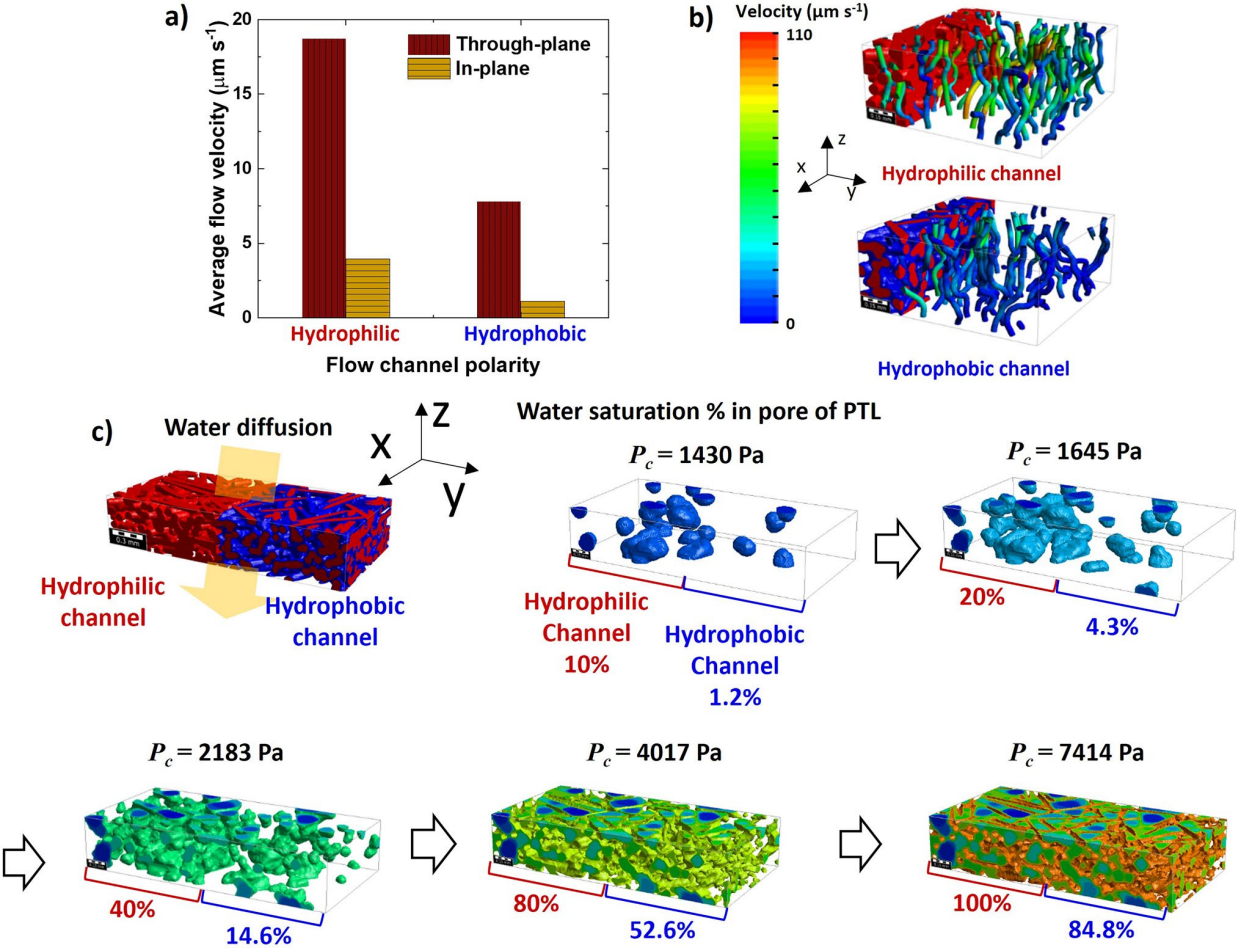
**a** Average flow velocity in the Ti PTL depending on polarity and **b** the corresponding through-plane flow stream images, obtained using the FlowDict module in GeoDict software. **c** Comparison of through-plane water saturation in the hydrophilic and hydrophobic channels with increasing capillary pressure (*P*_**c**_); these results are obtained using the GeoDict software with Imbibition

For further investigations, the GeoDict software was employed to simulate the flow dynamics in the PTLs by considering the surface polarity and porous structure based on the Ti PTL used in this study. A more detailed discussion on the mass flow is presented in Note S2. First, as summarized in Fig. 6a, the in-plane flow velocity (Fig. S5) is significantly lower than the through-plane velocity (Fig. 6b) in the PTL used in this study. Specifically, the in-plane flow velocity in the hydrophobic channel was calculated as 1.11 µm s^−1^. Thus, it is considerably lower than the velocities of the other flow components, which are 3.96 µm s^−1^ in the through-plane velocity of the hydrophobic channel and 7.82 and 18.72 µm s^−1^ in the in- and through-planes of the hydrophilic channel, respectively. Therefore, in the WE mode, where water supply is crucial, minimizing in-plane water diffusion in the hydrophobic channel is a key consideration, which is achieved in the SP configuration.

Second, as shown in Fig. 6c, the high surface energy of the hydrophilic channel effectively lowered the capillary pressure for penetration of water through the PTL. Consequently, in the FC mode, the generated water was efficiently transported via the hydrophilic channel, which simultaneously prevented water saturation in the CL and facilitated oxygen flow through the hydrophobic channel. This result supports the observation that the ${R}_{\text{mass}}$ values of the SP and R-SP are lower than that of the NP, as discussed above.

### Durability Test

**Fig. 7:**
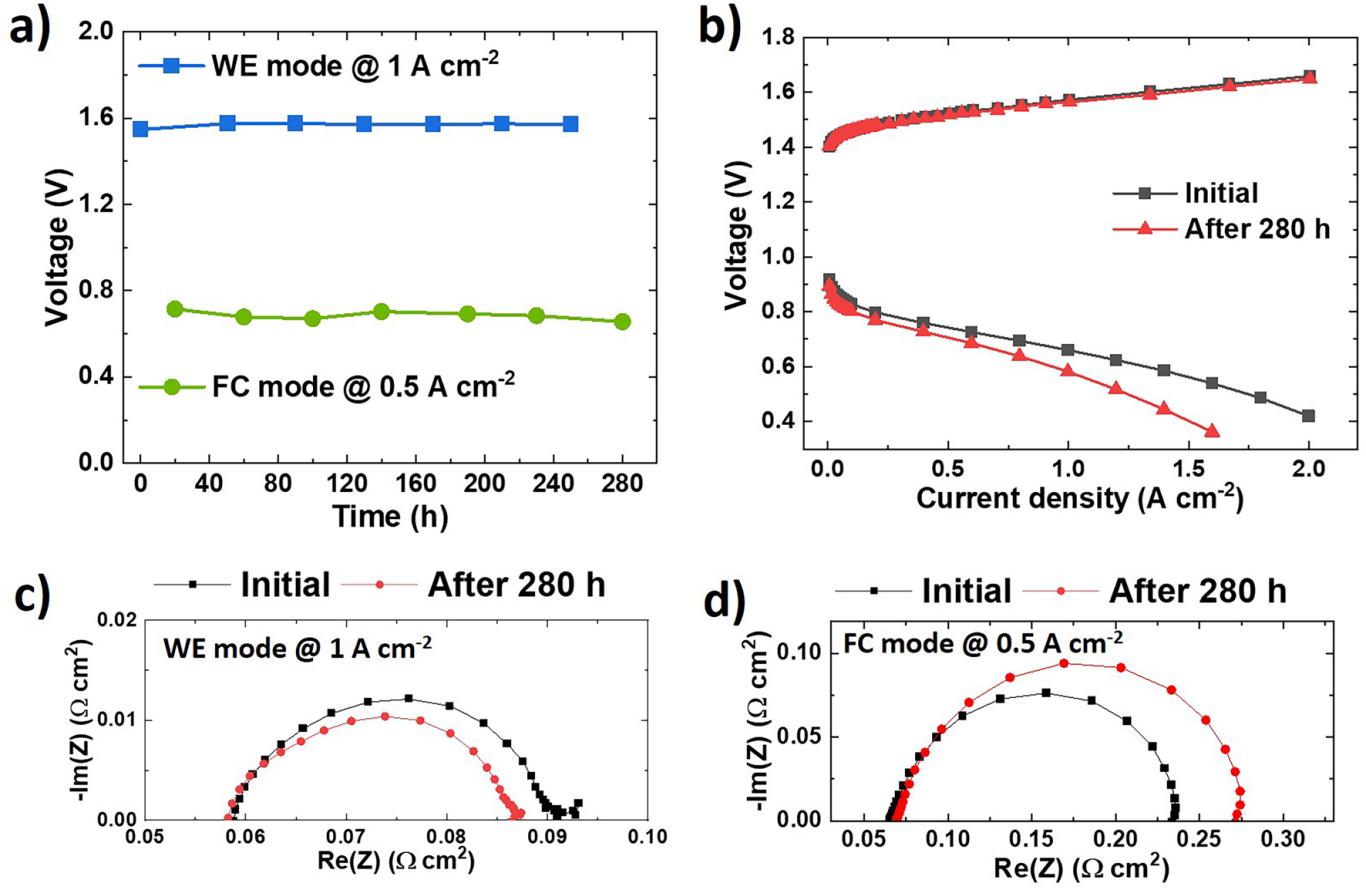
**a** Long-term operation of the unitized regenerative fuel cell device with the SP configuration at 1 and 0.5 A cm^−2^ in the WE and FC modes, respectively. **b** Polarization curves obtained before and after testing. The corresponding Nyquist plot in the **c** WE and **d** FC modes at 1 and 0.5 A cm^−2^, respectively

Finally, the long-term stability of the URFC was examined using the SP configuration for 280 h. The test involved seven cycles of WE and FC modes, with each mode being switched every 20 h, as shown in Fig. 7. Throughout the 280 h operation, the URFC demonstrated a stable performance, with small voltage degradation rates of 96.43 and − 210.7 μV h^−1^ in the WE and FC modes, respectively. However, a higher degradation in performance was noted in the FC mode compared to the WE mode (Fig. 7b). In contrast, the WE mode performance remained almost constant, and even slightly improved after the long-term test. This observation was primarily attributed to a change in the charge transport resistance (Fig. 7c, d).

**Fig. 8:**
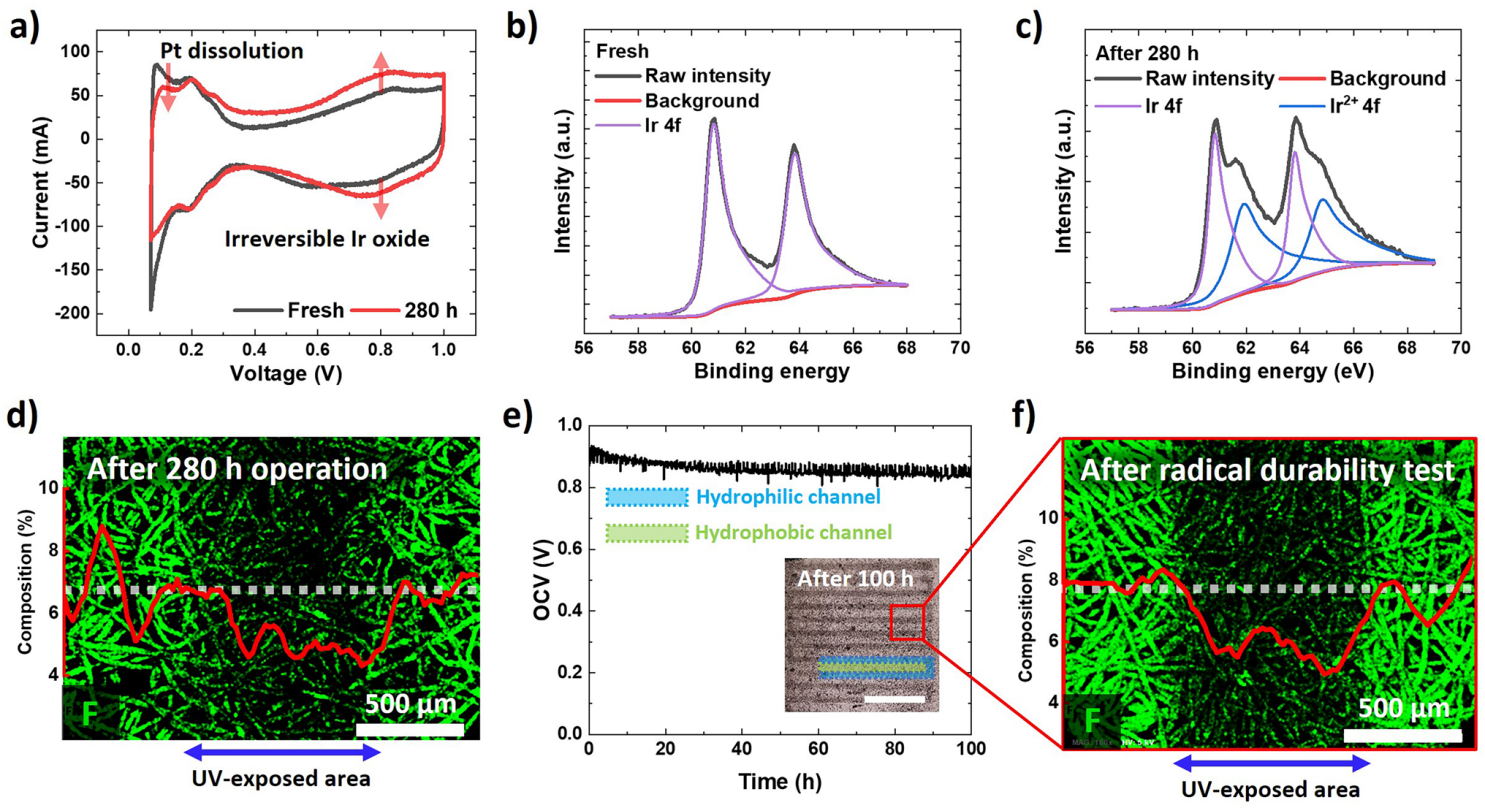
**a** Cyclic voltammograms of the SP configuration before and after testing. XPS profiles of the membrane electrode assembly **b** before and **c** after the long-term test for Ir 4*f* (oxygen electrode). **d** EDS mapping and line scan results of F after the long-term test. The radical durability test based on **e** an open-circuit-voltage hold test and **f** the corresponding EDS mapping and line scan results of F after testing

To investigate the cause of the performance degradation, the electrochemical surface area (ECSA) of the Pt catalyst in the oxygen electrode was examined using cyclic voltammetry. The ECSA of the Pt catalyst decreased by 27.8% after testing from 18.7 to 13.5 m^2^ g_Pt_^−1^ (Fig. 8a); whereas, the capacitance increased in the voltage window between 0.4 and 1 V because of the irreversible formation of Ir oxide [49, 50]. In addition, severe Pt catalyst loss was confirmed via X-ray photoelectron spectroscopy (XPS); whereas, Ir atoms were converted to their oxide forms and their composition increased (Figs. 8b, c, S6, and Table S2). Furthermore, agglomeration of the catalyst in the oxygen electrode, which is near the membrane, was observed after testing (Fig. S7). In contrast, a hydrophilic pattern in the SP exhibited a clear F gradient across the flow channel, with good durability during the 280 h operation (Fig. 4d) and even under the harsh radical-generating condition. During the open-circuit voltage hold test for 100 h at 90 °C and a relative humidity of 30% (Fig. 8e, f), the SP Ti PTL exhibited a distinctive F gradient between the hydrophilic and hydrophobic flow channels. Therefore, the voltage degradation in the FC mode was attributed to the ECSA loss of the Pt catalyst. Conversely, the decrease in the charge transport resistance observed in the WE mode was attributed to the recovery of the ECSA of the Ir catalyst, which was hindered by the Pt catalyst.

Based on the results obtained in previous studies, the severe degradation of the Pt catalyst in our URFCs was attributed to the dissolution of novel transition metals [51, 52]. During the round-trip operation of the URFCs, the catalyst surface undergoes repeated cycles of oxidation and reduction via mode switching. In the case of Pt and Ir, both PtO_*x*_ and IrO_*x*_ present on the catalyst’s surface are relatively stable and prevent catalyst dissolution under the oxidation potential. However, during the reduction process, a large amount of the catalyst is dissolved when the oxidation potential is switched to the reduction potential. Notably, in the voltage range of 0.6–1.6 V, the reduction of PtO_*x*_ to Pt is faster than that of IrO_*x*_ to Ir; and thus, the amount of Pt dissolved during this process is higher than that of Ir.

## Conclusion

We developed highly stable and efficient URFCs with optimized hydrophilic patterned Ti PTLs. Notably, the URFC with an SP configuration effectively regulated water path during the FC operation without compromising the WE performance. Moreover, it achieved an excellent RTE of 25.7% at 2 A cm^−2^, providing a high maximum power density of 0.87 W cm^−2^ in the FC mode. Our URFCs also exhibited a small voltage degradation over 280 h operation, and this voltage drop was primarily attributed to catalyst degradation. Our findings underscore the effectiveness of hydrophilic patterned Ti PTLs and the importance of optimizing their design with respect to the flow-field pattern of the BP to enhance the performance of URFCs significantly.

## Supplementary Information

Below is the link to the electronic supplementary material.

Supplementary file1 (AVI 20941 kb)

Supplementary file2 (AVI 14496 kb)

Supplementary file3 (DOCX 1175 kb)
