# Supplementary material for: Regulating Water Transport Paths on Porous Transport Layer by Hydrophilic Patterning for Highly Efficient Unitized Regenerative Fuel Cells
Source: Nanomicro Lett. 2025 Mar 17;17:189. doi: 10.1007/s40820-025-01684-6 (PMC11911311; doi:10.1007/s40820-025-01684-6)
Supplement: Supplementary file 3 — Supplementary file3 (DOCX 1175 kb) [file 40820_2025_1684_MOESM3_ESM.docx]

Supporting Information for

**Regulating Water Transport Paths on Porous Transport Layer by Hydrophilic Patterning for Highly Efficient Unitized Regenerative Fuel Cells**

Sung Min Lee^1^, Keun Hwan Oh^1^, Hwan Yeop Jeong^1^, Duk Man Yu^1^*, and Tae-Ho Kim^1^*

^1^Hydrogen Energy Research Center, Korea Research Institute of Chemical Technology (KRICT), 141 Gajeong-ro, Yuseong-gu, Daejeon, 305-600, Republic of Korea

*Corresponding authors. E-mail: [dmyu@krict.re.kr](mailto:dmyu@krict.re.kr) (Duk Man Yu); [thkim@krict.re.kr](mailto:thkim@krict.re.kr) (Tae-Ho Kim)

**Supplementary Figures and Tables**


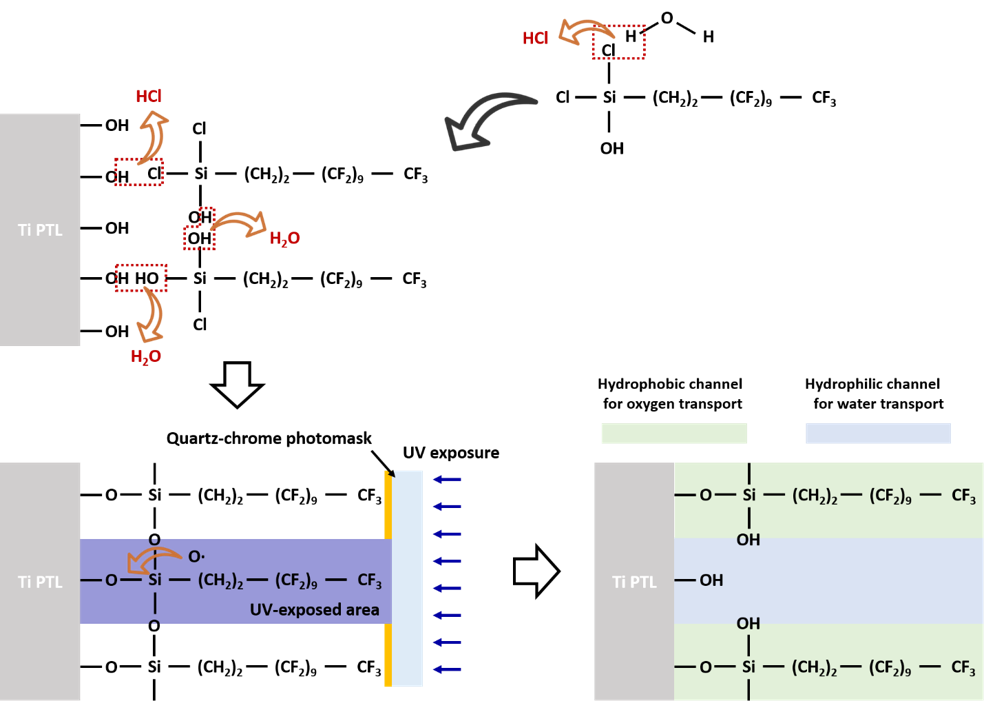


**Fig. S1** Schematic of the surface modification of Ti PTL with FDDTS


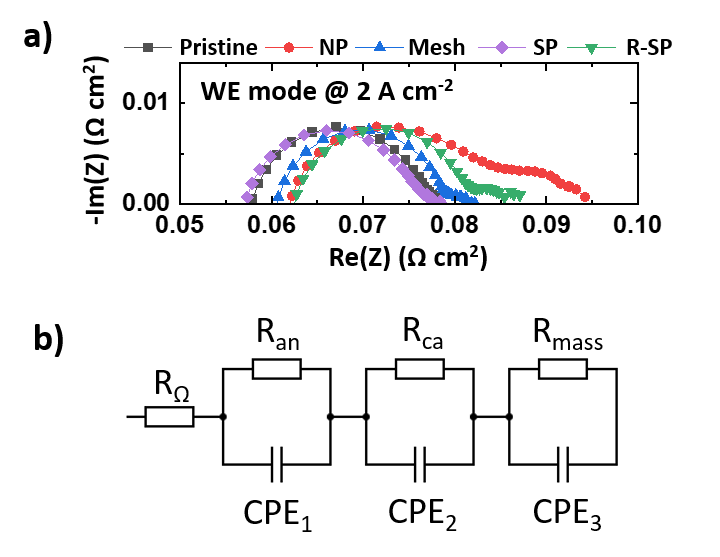


**Fig. S2** Nyquist plot obtained via EIS at 2 A cm^-2^ in the WE mode and b) corresponding equivalent circuit


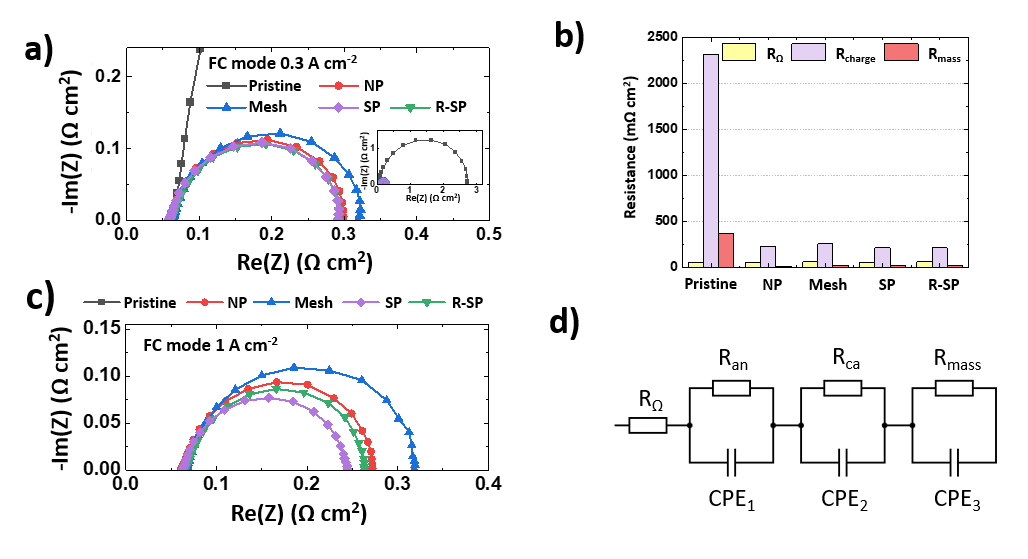


**Fig. S3** **a**) Nyquist plot obtained from EIS at 0.3 A cm^-2^ in the FC mode and **b**) corresponding resistance contributions. **c**) Nyquist plot obtained from EIS at 1 A cm^-2^ in the FC mode. **d**) Equivalent circuit for the Nyquist plot


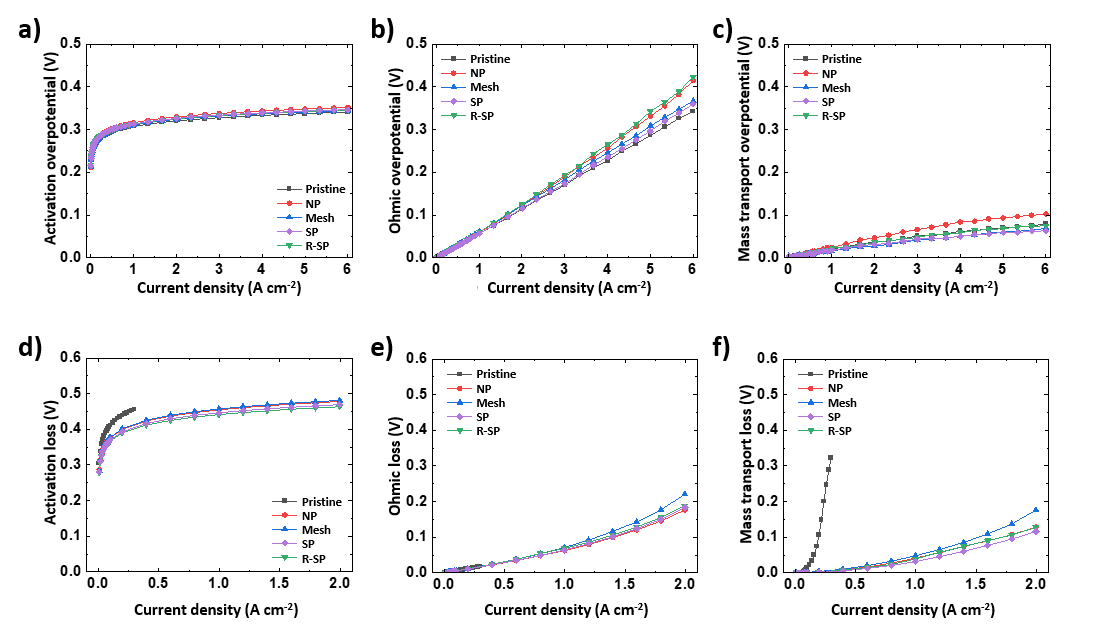


**Fig. S4** Overpotential in WE mode: **a**) activation, **b**) Ohmic, and **c**) mass transport overpotential. Voltage loss in FC mode: **d**) activation, **e**) Ohmic, and **f**) mass transport voltage loss


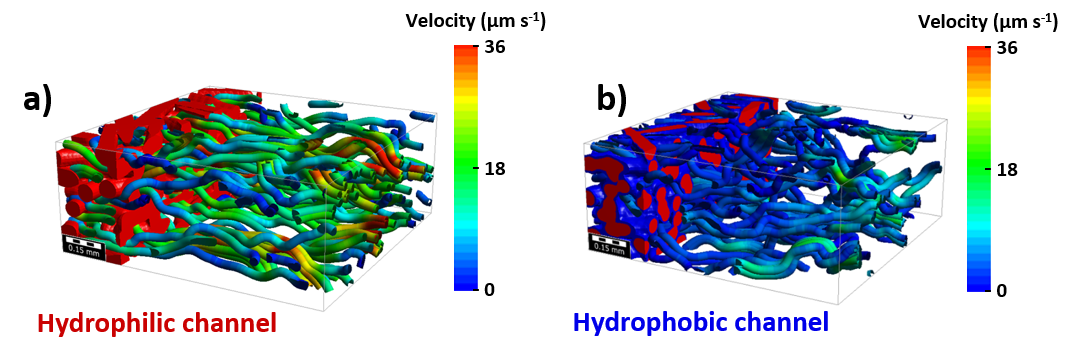


**Fig. S5** In-plane flow stream images obtained using the FlowDict module in GeoDict software


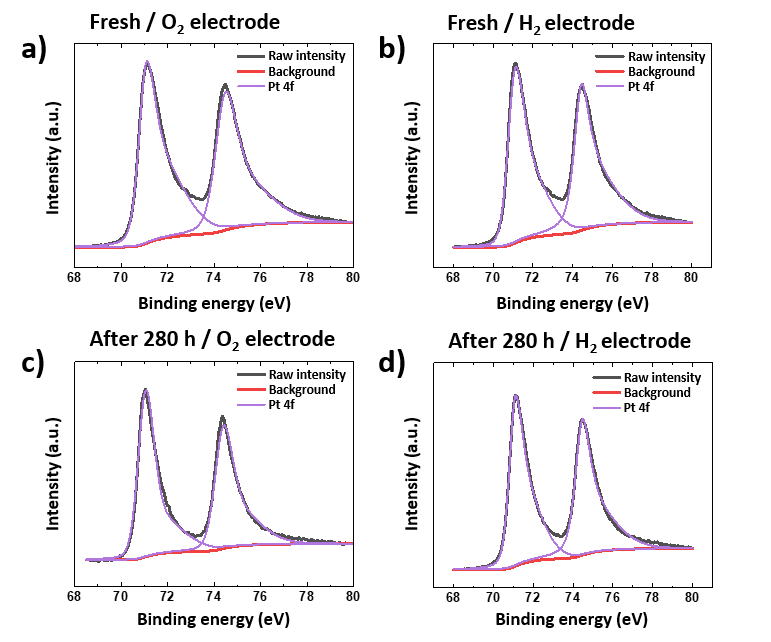


**Fig. S6** XPS profiles of fresh MEA for Pt 4f for **a**) oxygen and **b**) hydrogen electrodes. XPS profiles of MEA after the long-term test for Pt 4f for **c**) oxygen and d) hydrogen electrodes


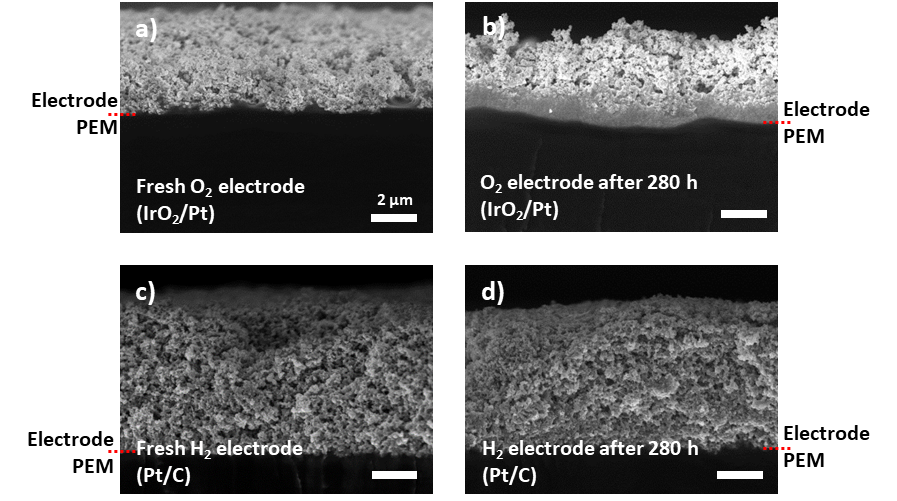


**Fig. S7** Cross-sectional field-emission SEM (FE-SEM) images of O_2_ electrode **a**) before and **b**) after the long-term tests. Cross-sectional FE-SEM images of H_2_ electrode **c**) before and **d**) after the long-term tests

**Note S1 UV/ozone patterning**

The process consists of two main steps. In the first step, FDDTS is deposited onto the Ti PTL using vapor-phase deposition [S1-S3]. During this deposition, a portion of the chloride in FDDTS reacts with atmospheric H_2_O, producing hydroxyl groups. The Si-Cl and Si-OH bonds in FDDTS further react with the hydroxyl groups on the Ti PTL surface, creating siloxane bonds between FDDTS and the Ti surface. Simultaneously, a condensation reaction occurs among the hydroxyl groups of the FDDTS molecules, forming densely packed, covalently bonded, hydrophobic self-assembled layers on the Ti PTL surface.

In the second step, the fluorosilanized Ti PTL is exposed to UV light in the presence of atmospheric oxygen through a quartz-chrome photomask. This exposure generates highly reactive gaseous oxygen radicals, exclusively within the UV-illuminated regions. These radicals then react with the FDDTS layers on the Ti PTL surface, resulting in the formation of hydroxyl groups [S4, S5]. Finally, the regions exposed to UV illumination develop hydrophilic water channels, while the remaining areas maintain their hydrophobic characteristics, creating distinct hydrophilic flow channels within the Ti PTL, regulating water transport path.

**Note S2 Mass flow analysis**

As observed in the results from the computational study (**Fig. 6** and **S5**), the hydrophobic channel requires a much higher capillary pressure for water saturation compared to the hydrophilic channel. This results in a significant reduction in the average water flow flux through the hydrophobic channel with a noticeable decrease, especially in in-plane water diffusion in the PTL. In contrast, oxygen gas can easily diffuse through both the hydrophobic channel and the unsaturated pores within the hydrophilic channel as it is less affected by surface polarity. Therefore, the discussion in this note focuses on water flow in relation to porosity saturation and its flow velocity in the hydrophobic/hydrophilic flow channels within the Ti PTL.

First, in the Pristine configuration, water can readily diffuse within the Ti PTL at high flow velocities of 7.82 µm s^-1^ in the in-plane direction and 18.72 µm s^-1^ in the through-plane direction (Fig. 6a, b). Furthermore, the flow channel can easily become saturated with water due to the lower capillary pressure for water saturation (Fig. 6c). Therefore, in the WE mode, the hydrophilic nature of the pristine Ti PTL facilitates efficient water supply. However, simultaneously, the Pristine configuration is expected to experience interference between water inflow and gas outflow due to the scarcity of vacancies for gas passage. Consequently, the actual flow velocity of water in the Pristine configuration is anticipated to decrease compared to the calculated value (**Fig. 4**c) in the WE mode. Conversely, when switched to the FC mode, the water-saturated Ti PTL disturbs oxygen diffusion toward the catalyst layer. As a result, allowing oxygen gas to pass through the water-saturated Ti PTL requires high pressure to overcome the capillary pressure of water, resulting in a significant reduction in gas diffusion toward the catalyst layer.

In contrast, the NP configuration, composed entirely of the hydrophobic channel, exhibits extremely low water flow velocity (1.11 µm s^-1^ in the in-plane direction and 3.96 µm s^-1^ in the through-plane direction; Fig. 6a). Additionally, as illustrated in **Fig. S5**, the hydrophobic channel displays discontinuous water flow streamlines compared to the hydrophilic channel. This indicates that water diffusion within the hydrophobic channel is considerably sluggish, creating numerous vacancies. Consequently, in the WE mode, water supply to the catalyst layer is significantly impeded, resulting in a surplus of vacancies (Fig. 4d). This, in turn, leads to an increase in both ohmic resistance and mass transport resistance, resulting in a severe decrease in WE performance. However, when it switches to the FC mode, similar to a conventional FC configuration, sufficient porosity is guaranteed for oxygen supply due to the low water saturation level of the hydrophobic channel, enabling efficient mass transport in the FC mode. Water generated during FC operation must simultaneously overcome the high capillary pressure of the hydrophobic channel to pass through the PTL. Therefore, the catalyst layer can be saturated by water, reducing oxygen availability (**Fig. 5**b, d).

In the case of the R-SP configuration, the hydrophobic channel is directly aligned with the BP's flow field. Consequently, during the WE mode, the water stream must pass through the hydrophobic channel to reach the hydrophilic channel, with an extremely slow in-plane flow velocity of 1.11 µm s^-1^. Nevertheless, unlike in the NP configuration, the hydrophilic channel in the R-SP configuration contributes to the reduction of mass transport resistance (R_Ω_ = 15.35 Ω cm^2^ for NP, 8.05 Ω cm^2^ for R-SP). As a result, the WE performance of the R-SP configuration shows a small improvement compared to the NP configuration. In the FC mode with the R-SP configuration, the mass transport resistance is measured at 30.47 Ω cm^2^, slightly lower than the 43.10 Ω cm^2^ observed in the NP configuration. This suggests that in the R-SP configuration, the hydrophilic channel effectively facilitated the removal of generated water by reducing the capillary pressure in diffusion across the PTL for water, preventing water saturation in the catalyst layer (**Fig. 6**).

In the case of the Mesh and SP configurations, where the hydrophilic channel is partially aligned (Mesh) and properly aligned (SP) with the flow field of BP, efficient water diffusion in the WE mode was facilitated through the hydrophilic channel. In these configurations, the unfavorable slow diffusion of water in the hydrophobic channel was minimized, resulting in a significant decrease in mass transport resistance. Particularly, the SP configuration exhibited the lowest ohmic resistance of 57.20 Ω cm^2^, lower than the 57.85 Ω cm^2^ of the Pristine configuration, while the mass transport resistance was comparable to the Pristine configuration (3.25 Ω cm^2^ for SP, 3.05 Ω cm^2^ for Pristine). This implies that although the hydrophobic channel in the SP configuration slightly reduced the available water diffusion channel area in the PTL, the high flow velocity can be developed in the hydrophilic channel of SP Ti PTL, minimizing interference between water inflow and gas outflow (**Fig. 4f**). Therefore, the effective removal of both oxygen gas and water supply is facilitated in the SP configuration, resulting in the compensation for reduced hydrophilic channel ratio in SP Ti PTL. Conversely, in the FC mode with the Mesh configuration, relatively higher voltage degradation is observed than in the other configurations (**Fig. 5a**). In the Mesh configuration, the in-plane diffusion of gas in Ti PTL near the MEA is relatively restricted due to the water-saturated hydrophilic channel configured in a mesh pattern, resulting in poor in-plane oxygen distribution in the catalyst layer (**Fig. 5e**). Therefore, the non-uniform gas distribution results in a performance degradation in the Mesh configuration. Unlike the case of the Mesh configuration, in the SP configuration, in-plane gas diffusion is less restricted due to the pattern geometry. In addition, the direct alignment of the hydrophilic channel with the BP's flow field in the SP configuration effectively contributes to the removal of water from the CL. Simultaneously, gas flow can easily diffuse into the hydrophobic channel passing through the hydrophilic channel near the BP, as the water is removed to the BP's flow field, creating sufficient pores for gas diffusion. Therefore, both the charge and mass transport resistances can be minimized in the SP configuration among these five configurations: Pristine, NP, Mesh, SP, and R-SP.

**Table S1** Comparison of round-trip efficiency and detailed operation condition of recently reported works [S6-S13]


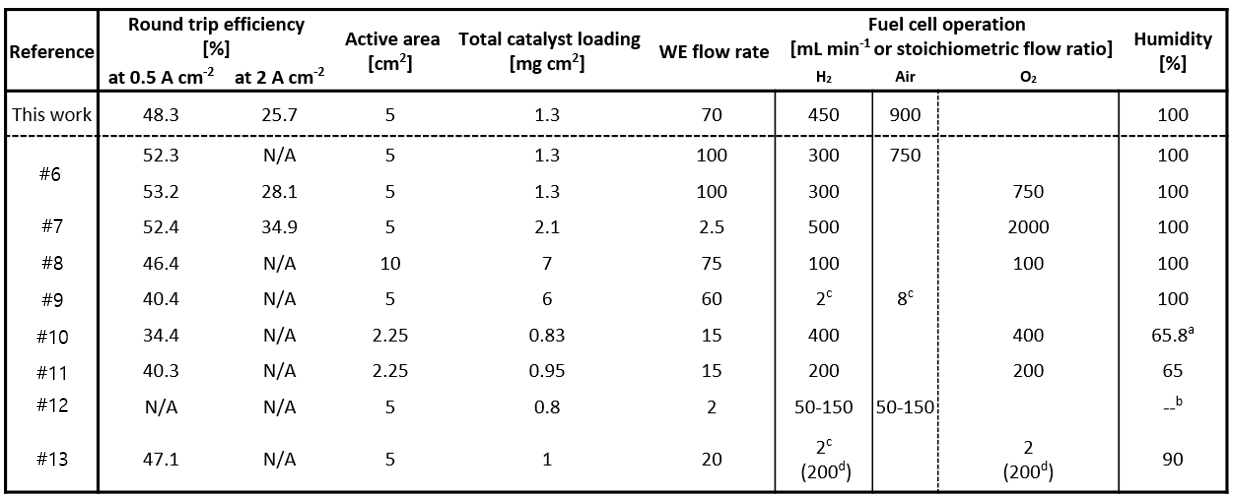


^a^ Calculated from humidifier temperature

^b^ Not stated

^c^ Stoichiometric flow ratio

^d^ Minimum flow rate

**Table S2** Atomic compositions from XPS


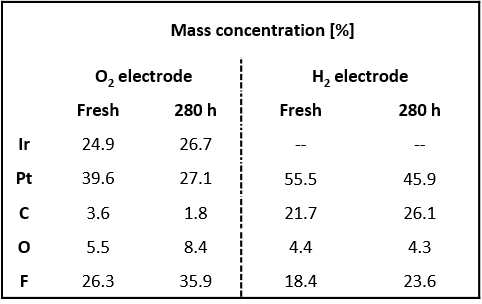


**Table S3** Various parameters for GeoDict simulations


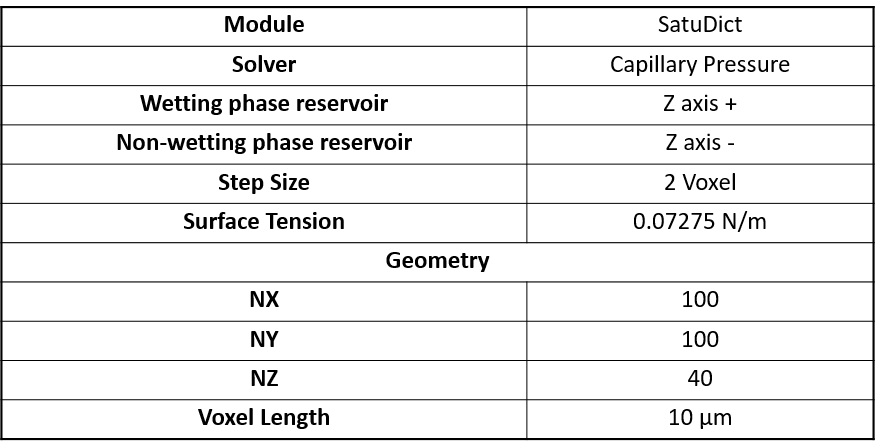


**Movie S1** Gas flow through the hydrophobic channel in the Mesh Ti PTL

N_2_ gas was supplied from the bottom, penetrating the Mesh Ti PTL. To visualize gas flow, the Mesh Ti PTL was submerged in deionized water. In Movie S1, the hydrophilic channel is indicated by the dashed line, while the remainder of the area represents the hydrophobic channel in the Mesh Ti PTL. Gas bubbles were predominantly observed on the surface of the hydrophobic channel, indicating that gas is transported through the empty pores in the hydrophobic channel when the hydrophilic channel is fully wet.

**Movie S2** Water flow through the hydrophilic channel in the Mesh Ti PTL

Deionized water was supplied from the bottom, penetrating the Mesh Ti PTL. In Movie S2, the hydrophilic channel is indicated by the dashed line, while the remaining area represents the hydrophobic channel in the Mesh Ti PTL. Initially, bubbles of water were predominantly observed on the surface of the hydrophilic channel, indicating that water is primarily transported through the hydrophilic channel.

**Supplementary References**

1. S. Oyola-Reynoso, Z. Wang, J. Chen, S. Çınar, B. Chang et al., Revisiting the challenges in fabricating uniform coatings with polyfunctional molecules on high surface energy materials. Coatings **5**, 1002–1018 (2015). <https://doi.org/10.3390/coatings5041002>
2. D. K. Aswal, S. Lenfant, D. Guerin, J. V. Yakhmi, D. Vuillaume, Self assembled monolayers on silicon for molecular electronics. Anal. Chim. Acta **568**, 84–108 (2006). <https://doi.org/10.1016/j.aca.2005.10.027>
3. S. Oyola-Reynoso, I. Tevis, J. Chen, J. F. Bloch, M. Thuo, Fabrication of Namib beetle inspired biomimetic amphi-phobic surfaces using adsorbed water as a monomer. Procedia Eng. **141**, 59–62 (2016). <https://doi.org/10.1016/j.proeng.2015.08.1105>
4. S. M. Lee, S. Oh, S. T. Chang, Highly transparent, flexible conductors and heaters based on metal nanomesh structures manufactured using an all-water-based solution process. ACS Appl. Mater. Interfaces **11**, 4541–4550 (2019). <https://doi.org/10.1021/acsami.8b17415>
5. S. M. Lee, S. K. Song, S. Yoon, D. S. Chung, S. T. Chang, Liquid thin film dewetting-driven micropatterning of reduced graphene oxide electrodes for high performance OFETs. J. Mater. Chem. C **7**, 153–160 (2019). [https://doi.org/10.1039/C8TC03981J](https://doi.org/10.1039/c8tc03981j)
6. X. Peng, Z. Taie, J. Liu, Y. Zhang, X. Peng et al, Hierarchical electrode design of highly efficient and stable unitized regenerative fuel cells (URFCs) for long-term energy storage. Energy Environ. Sci. **13**, 4872–4881 (2020). [https://doi.org/10.1039/D0EE03244A](https://doi.org/10.1039/d0ee03244a)
7. S. Komini Babu, A. Yilmaz, M. A. Uddin, J. LaManna, E. Baltic et al., A goldilocks approach to water management: Hydrochannel porous transport layers for unitized reversible fuel cells. Adv. Energy Mater. **13**, 2203952 (2023). <https://doi.org/10.1002/aenm.202203952>
8. T. Ioroi, T. Oku, K. Yasuda, N. Kumagai, Y. Miyazaki, Influence of PTFE coating on gas diffusion backing for unitized regenerative polymer electrolyte fuel cells. J. Power Sources **124**, 385–389 (2003). [https://doi.org/10.1016/S0378-7753(03)00795-X](https://doi.org/10.1016/s0378-7753(03)00795-x)
9. A. Rocha, R. B. Ferreira, D. S. Falcão, A. M. F. R. Pinto, Experimental study on a unitized regenerative fuel cell operated in constant electrode mode: Effect of cell design and operating conditions. Renew. Energy **215**, 118870 (2023). <https://doi.org/10.1016/j.renene.2023.05.128>
10. A. Lim, J. Kim, H. J. Lee, H.-J. Kim, S. J. Yoo et al., Low-loading IrO_2_ supported on Pt for catalysis of PEM water electrolysis and regenerative fuel cells. Appl. Cat. B **272**, 118955 (2020). <https://doi.org/10.1016/j.apcatb.2020.118955>
11. A. Lim, H.-Y. Jeong, Y. Lim, J. Y. Kim, H. Y. Park et al., Amphiphilic Ti porous transport layer for highly effective PEM unitized regenerative fuel cells. Sci. Adv. **7**, eabf7866 (2021). <https://doi.org/10.1126/sciadv.abf7866>
12. J. C. Cruz, V. Baglio, S. Siracusano, R. Ornelas, L. G. Arriaga et al., Nanosized Pt/IrO_2_ electrocatalyst prepared by modified polyol method for application as dual function oxygen electrode in unitized regenerative fuel cells. Int. J. Hydrog. Energy **37**, 5508–5517 (2012). <https://doi.org/10.1016/j.ijhydene.2011.12.153>
13. P. Gayen, X. Liu, C. He, S. Saha, V. K. Ramani, Bidirectional energy & fuel production using RTO-supported-Pt–IrO_2_ loaded fixed polarity unitized regenerative fuel cells. Sustainable Energy Fuels **5**, 2734–2746 (2021). [https://doi.org/10.1039/D1SE00103E](https://doi.org/10.1039/d1se00103e)
